# Intraoperative Resting-State Functional Connectivity Based on RGB Imaging

**DOI:** 10.3390/diagnostics11112067

**Published:** 2021-11-09

**Authors:** Charly Caredda, Laurent Mahieu-Williame, Raphaël Sablong, Michaël Sdika, Fabien C. Schneider, Jacques Guyotat, Bruno Montcel

**Affiliations:** 1 INSA-Lyon, Univ Lyon, Université Claude Bernard Lyon 1, UJM-Saint Etienne, CNRS, Inserm, CREATIS UMR 5220, U1206, F69100 Lyon, France; Laurent.Mahieu-Williame@creatis.insa-lyon.fr (L.M.-W.); Raphael.Sablong@creatis.insa-lyon.fr (R.S.); Michael.Sdika@creatis.insa-lyon.fr (M.S.); 2 Service de Radiologie, Centre Hospitalier Universitaire de Saint Etienne, TAPE EA7423, Université de Lyon, UJM, F42023 Saint Etienne, France; Fabien.Schneider@univ-st-etienne.fr; 3 Service de Neurochirurgie D, Hospices Civils de Lyon, F69500 Bron, France; jacques.guyotat@chu-lyon.fr

**Keywords:** resting-state, functional connectivity, intraoperative imaging, optical imaging, RGB imaging

## Abstract

RGB optical imaging is a marker-free, contactless, and non-invasive technique that is able to monitor hemodynamic brain response following neuronal activation using task-based and resting-state procedures. Magnetic resonance imaging (fMRI) and functional near infra-red spectroscopy (fNIRS) resting-state procedures cannot be used intraoperatively but RGB imaging provides an ideal solution to identify resting-state networks during a neurosurgical operation. We applied resting-state methodologies to intraoperative RGB imaging and evaluated their ability to identify resting-state networks. We adapted two resting-state methodologies from fMRI for the identification of resting-state networks using intraoperative RGB imaging. Measurements were performed in 3 patients who underwent resection of lesions adjacent to motor sites. The resting-state networks were compared to the identifications provided by RGB task-based imaging and electrical brain stimulation. Intraoperative RGB resting-state networks corresponded to RGB task-based imaging (DICE:0.55±0.29). Resting state procedures showed a strong correspondence between them (DICE:0.66±0.11) and with electrical brain stimulation. RGB imaging is a relevant technique for intraoperative resting-state networks identification. Intraoperative resting-state imaging has several advantages compared to functional task-based analyses: data acquisition is shorter, less complex, and less demanding for the patients, especially for those unable to perform the tasks.

## 1. Introduction

Non-invasive functional brain mapping is an imaging technique that allows the locating of functional areas of the patient’s brain. This technique is used during brain tumor resection surgery to indicate to the neurosurgeon the cortical tissues which should not be removed without cognitive impairment. Functional magnetic resonance imaging (fMRI) [1] is the preoperative gold standard for identifying the patient’s functional areas. However, after the patient’s craniotomy, a brain shift invalidates the relevance of neuronavigation to localize functional areas during surgery [2]. To avoid localization errors, intraoperative MRI has been suggested, but it complicates the surgical procedure and is, therefore, rarely used. For these reasons, electrical brain stimulation (EBS) [3] is preferred during neurosurgery, but this technique is mainly limited by its low spatial resolution (≈5 mm [4]) and has the potential risk to trigger epileptic seizures. Optical imaging provides an ideal solution for intraoperative functional brain mapping because the analysis of the light absorption allows to monitor the brain activity (motor or sensory tasks for example) with quantification of the concentration changes in oxy- ($\Delta C_{HbO_{2}}$) and deoxygenated hemoglobin ($\Delta C_{Hb}$) in brain cortex [5,6,7,8,9,10,11,12].

As opposed to functional task-based analyses, resting-state functional connectivity aims to identify the low frequency cortical hemodynamic fluctuations (<0.1 Hz) that reflect the patient neuronal activity at rest and that are linked to resting-state networks [13]. These fluctuations can occur in the absence of a task, and are often correlated between functionally related areas. Resting-state imaging has several advantages compared to functional task-based analyses: data acquisition is shorter, less complex, and less demanding for the patients, especially for those unable to perform the tasks. This technique is widely used in fMRI studies [14,15,16,17], could be used to plan tumor resection [15] when patients are under general anesthesia [18,19]. Using optical imaging, resting-state techniques have been adapted to functional near-infrared spectroscopy (fNIRS) devices [20,21] for continuous bedside monitoring and wide field devices for studying neurovascular coupling in mice brains [22,23]. To our knowledge, intraoperative implementation of optical resting-state has never been proposed in the literature. Main issues come from the partial access to the brain cortex, whereas the models of resting-state used whole brain imaging.

In these works, we demonstrate that intraoperative optical resting-state can be implemented intraoperatively using two standard fMRI resting state techniques (the seed correlation and the independent component analyses [13]). The cortical areas identified with optical resting-state maps correspond to those identified by optical functional task-based analysis and EBS [3] with patients who are awake or under general anesthesia. These results could help to guide the neurosurgeon surgical gesture, have the potential to reduce the duration of surgical operations while improving patient and neurosurgeon comfort.

## 2. Material and Methods

### 2.1. Intraoperative Procedure

The study was conducted at the neurological center of the Pierre Wertheimer hospital in Bron, France. Three patients presenting a tumor close to the motor cortex area were included in the study. All experiments were approved by the local ethics committee of Lyon University Hospital (France) and the participating patients signed written consent. The videos were acquired with the wide field optical device described in [7] after the patient’s craniotomy and before the brain tumor resection surgery. For each patient, resting-state and task-based data were acquired. For task-based data, motor cortex stimulation was performed by repetitive, alternating hand opening and closing at ≈1 Hz (successive periods of 20 s of rest followed by 20 s of stimulation). For resting-state data, patients stayed at rest and the acquisition duration was at least 1 min 40 s, which corresponds to the minimum acquisition time required to obtain accurate and stable mapping of brain connectivity network in children using fNIRS [24] and in adult using fMRI [25]. Information on patients and acquisitions is summarized in Table 1.

The neurosurgeon performed EBS after RGB imaging using a bipolar electrode (Nimbus Medtronic neurostimulator). A biphasic current was used (pulsating frequency: 60 Hz, pulse width: 1 ms). The current was first set to 1 mA, and increased to 6 mA. When a functional area was identified by EBS, the neurosurgeon placed a colored pastille on the patient cortex and a RGB image was acquired to store the position of the functional area in the RGB image.

### 2.2. Functional Analyses

**Figure 1:**
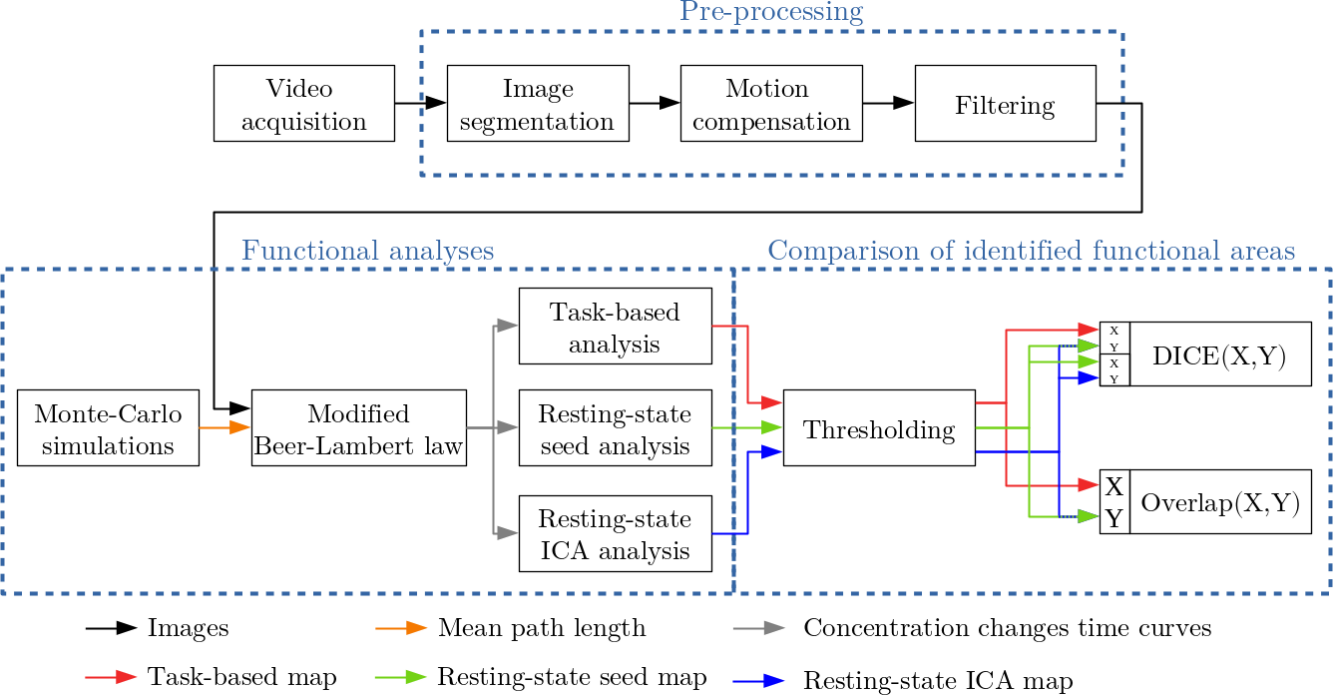
Overview of the algorithm [7] for the computation of concentration changes time curves, the calculation of functional maps (task-based and resting-state maps) and the comparison of functional areas identified by functional maps.

Once the video was acquired, the quantitative model described in [7] was applied. A schematic overview of these processing steps is represented in Figure 1.

The first image of the video sequence was segmented into three classes: gray matter, surface blood vessel, and buried blood vessel. The objective is to use in the modified Beer–Lambert law, and this for each pixel of the camera, the appropriate mean path length of photons travelled in tissue. For this purpose Monte Carlo simulations were performed using MCX software [26]. For each frame of the video, the repetitive brain motion was compensated [27,28]. The slow drift of RGB intensities was corrected due to tissue desiccation [29] and a low-pass filtering was performed to isolate slow hemodynamic fluctuations (cut-off frequency: 0.08 Hz [13,20,21]). Then, $\Delta C_{HbO_{2}}$ and $\Delta C_{Hb}$ time curves were computed for each camera pixel using the modified Beer–Lambert law [7,8]. To compute these concentration changes time curves, a software developed in C++ was used. This software is based on the Qt framework (v5.9.4) and open source libraries (OpenCV (v3.2.0) [30] and FFTW (v3.3.7) [31]). Then, several analyses were performed to identify functional brain maps. A task-based (see Section 2.2.1) and resting-state analyses (see Section 2.2.2) were computed. The extent of functional brain areas was identified with a thresholding operation (see Section 2.2.3). Finally, the binary functional maps were compared to each other with the calculation of the DICE and overlap coefficients (see Section 2.2.3).

#### 2.2.1. Task-Based Functional Analysis

The task-based analysis consisted in analyzing the correlation between measured and theoretical cortical hemodynamic changes. The theoretical hemodynamic time curve (*H*) was obtained by convolving the hemodynamic impulse response function [32] to a rectangular function that represented the patient’s physiological events (0: rest, 1: stimulation). Theoretical $\Delta C_{HbO_{2}}$ and $\Delta C_{Hb}$ time curves were obtained by multiplying *H* by 1 and −1, respectively. The Pearson correlation coefficient was computed between the theoretical and measured $\Delta C_{HbO_{2}}$ and $\Delta C_{Hb}$ time curves to produce task-based maps. For this analysis, the C++ software was used.

#### 2.2.2. Resting-State Analyses

The seed correlation [20] analysis and the independent component analysis (ICA) [33,34] are the main resting-state functional connectivity analyses.

The seed correlation analysis analyzes the correlation between measured $\Delta C_{HbO_{2}}$ and $\Delta C_{Hb}$ time curves and those measured at the level of a seed region. The seed was represented as a 20 pixels diameter disk (1.2 mm diameter) located on the motor area identified by EBS. $\Delta C_{HbO_{2}}$ and $\Delta C_{Hb}$ time curves measured at the level of the seed were averaged over its surface and were compared to the other time curves measured on the surface of the patient’s cortex using the Pearson correlation coefficient to produce two resting-state seed maps. For this analysis, the C++ software was used.

The ICA identifies original signals from a mixture of signals by assuming that the original signals are independent of each other. In our analysis, the original signal was $\Delta C_{n}$ (*n*: $HbO_{2}$ or Hb) and had a dimension of P×T (with *P* the number pixels of the image and *T* the number of frames acquired). Input data were normalized as follow:

(1) $$\Delta C_{n}^{\prime }\left(p,t\right)=\frac{\Delta C_{n}\left(p,t\right)-\mu_{n}\left(p\right)}{\sigma_{n}\left(p\right)},$$

where $\mu_{n}\left(p\right)$ and $\sigma_{n}\left(p\right)$ are the temporal mean and standard deviation of $\Delta C_{n}$ measured for the pixel *p* respectively. The normalization transforms the temporal vector $\Delta C_{n}\left(p\right)$ to $\Delta C_{n}^{\prime }\left(p\right)$ which is zero-mean and has unit variance. The matrix $\Delta C_{n}^{\prime }$ can be expressed as a linear combination of *K* sources:

(2) $$\Delta C_{n}^{\prime }=A\times S.$$

*S* is a matrix of dimension K×T and denotes the concentration changes sources in the patient brain. *A* is the mixing matrix of dimension P×K that express the spatial distribution of the sources. ICA decomposition leads to the estimation of the *K* sources:

(3) $$\tilde{S}=W\times \Delta C_{n}^{\prime }.$$

$\tilde{S}$ is the estimated sources (dimension K×T) and *W* is an unmixing matrix of dimension K×P. The spatial distribution of the sources *A* can be estimated by calculating the pseudo-inverse of the matrix *W*.

Using the FastICA algorithm [35] from the scikit-learn Python library (v0.18.1) [36], the normalized concentration changes matrix was decomposed into *K* independent sources. The number of independent components depends on the size of analyzed data. Between 10 and 20 independent components were used to analyze data acquired by a high-density diffuse optical tomography device [33]. Since our field of view was smaller and as only the sensorimotor function was exposed, we have chosen a smaller number of independent sources: K=5. The estimated matrix *A* ($\tilde{A}$) was reconstructed into 5 images to illustrate the spatial distribution of hemodynamics fluctuations sources. The 5 images were sorted by their variance (from the smallest to the largest value).

#### 2.2.3. Comparison of Identified Functional Areas

The task-based maps were compared to the resting-state maps to evaluate the identification performance of cortical functional zones. For this purpose, the NumPy Python library (v1.19.4) [37] was used. Each task-based and resting-state image was thresholded to obtain a binary image. The threshold value *T* was applied to the images:

(4) $$T=\left\{\begin{array}{cc} \mu_{I}+\alpha .\sigma_{I} & \mathrm{if}\;\mu_{I}\geq 0\\ \mu_{I}-\alpha .\sigma_{I}, & \mathrm{otherwise} \end{array}\right.$$

$\mu_{I}$ is the mean value of the image *I*, $\sigma_{I}$ its standard deviation and α∈[0;1] is the severity criterion of the thresholding operation equal to 0.75. Morphological opening and closing operations were applied to the binary image to remove isolated pixels and to close holes. A 20 pixel wide circular structuring element was used.

The DICE coefficient [38] was computed between binary task-based (*X* in Equation (5)) and resting-state seed or ICA maps (*Y* in Equation (5)). We also computed this metric between binary resting-state seed (*X* in Equation (5)) and ICA maps (*Y* in Equation (5)):

(5) $$DICE\left(X,Y\right)=\frac{2|X\;\cap \;Y|}{\left|X|+|Y\right|},$$

where |X| and |Y| are the cardinalities of the two sets calculated after the thresholding operation (see Equation (4)).

Resting-state fMRI is likely to reveal the whole motor cortex, whereas a single body segment (e.g., face, hand) would be obtained with task-based fMRI. To evaluate this case, the overlap coefficient [15] was computed between binary task-based (*X* in Equation (6)) and resting-state seed or ICA maps (*Y* in Equation (6)).

(6) $$Overlap\left(X,Y\right)=\frac{\left|X\;\cap \;Y\right|}{\left|X\right|},$$

where |X| and |Y| are the cardinalities of the two sets calculated after the thresholding operation (see Equation (4)).

Finally, we tested if the functional brain areas identified by EBS corresponded to the identifications provided by the task-based and resting-state maps by testing if EBS results were included in the binary task-based and resting-state maps.

## 3. Results

**Figure 2:**
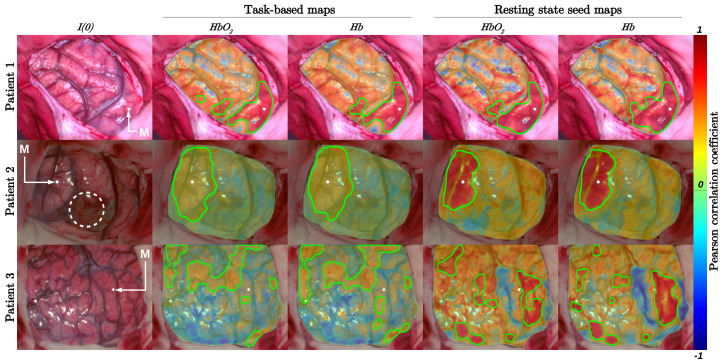
In the first column, the first image of the video sequence acquired for the three patients was represented (I(0)). In the second and third columns, the $HbO_{2}$ and Hb task-based maps were plotted, respectively. In the fourth and fifth columns, the $HbO_{2}$ and Hb resting-state seed maps were plotted, respectively. The seeds used for the computation of the resting-state maps were indicated by white spots and were located at the level of the motor area identified by electrical brain stimulation (letter M). The colorbar indicated the Pearson correlation coefficient values computed for each pixel. The green contours plotted in task-based and resting-state maps delimited the extent of the thresholded images (see Equation (4)). For patient 2 maps, the dotted white circle delimited the patient’s tumor. For patients 1 and 3, tumors were not observable on optical images.

**Figure 3:**
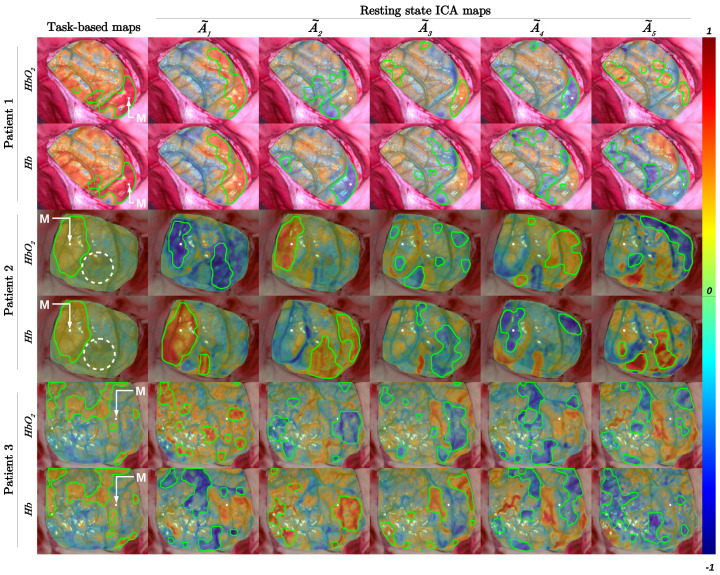
In the first column, the task-based maps were plotted. The spatial distribution of $\Delta C_{HbO_{2}}$ and $\Delta C_{Hb}$ fluctuations identified by ICA were represented in other columns. In columns 2 to 6, matrices $\tilde{A_{1}}$ to $\tilde{A_{5}}$ were represented for each chromophore and for each patient. Each motor area identified by electrical brain stimulation was indicated by a white spot and by the letter M. The colorbar indicated the range of variation of the Pearson correlation coefficient (task-based maps) and the range of variation of the ICA matrices. The resting-state ICA matrices were normalized with respect to the absolute value of their maximum, keeping zero in the center of the color scale. The green contours plotted in task-based and resting-state maps delimited the extent of the thresholded images (see Equation (4)). For patient 2 maps, the dotted white circle delimited the patient’s tumor. For patients 1 and 3, tumors were not observable on optical images.

The task-based and resting-state maps computed for the three patients are represented in Figure 2 and Figure 3. The seed and ICA analysis were used in Figure 2 and Figure 3, respectively. Each motor area identified by EBS was indicated by a white spot and the letter M. The white spot also indicated the seed area used for the resting-state seed analysis. The areas delimited by green contours indicated the contours of the binary task-based and resting-state maps obtained after the thresholding operations, see Equation (4). In Figure 2, the colorbar indicated the range of variation of Pearson correlation coefficients. In Figure 3, it illustrated the spatial distribution of hemodynamic fluctuations in ICA images. The values of the DICE and overlap coefficients computed between the task-based and resting-state seed maps are indicated in Table 2. The values of the DICE and overlap coefficients computed between the task-based and resting-state ICA binary maps and the DICE coefficients computed between the resting-state seed and ICA binary maps are indicated in Table 3.

For patient 1 and 2, the task-based maps corresponded to the resting-state seed maps (0.77≤DICE≤0.85) but were not entirely included in the resting-state seed maps (0.64≤Overlap≤0.89). For patient 3, the task-based masks did not match the resting-state seed maps (DICE≤0.12) and were hardly included in the resting-state seed maps (Overlap≤0.11).

For patient 1, on images $\tilde{A_{1}}$ ($HbO_{2}$ and Hb), $\tilde{A_{2}}$ (Hb), $\tilde{A_{3}}$ ($HbO_{2}$), and $\tilde{A_{4}}$ ($HbO_{2}$), the binary masks corresponded to those identified on the task-based maps (0.49≤DICE≤0.74 and 0.46≤Overlap≤0.70). When comparing resting-state seed and ICA images, the strongest DICE coefficients were obtained with the images $\tilde{A_{4}}$ for $HbO_{2}$ (DICE=0.52) and $\tilde{A_{2}}$ for Hb (DICE=0.70). For patient 2, on images $\tilde{A_{1}}$ ($HbO_{2}$ and Hb), $\tilde{A_{2}}$ ($HbO_{2}$) and $\tilde{A_{4}}$ (Hb), the binary masks corresponded to those identified on the task-based maps (0.47≤DICE≤0.75 and 0.41≤Overlap≤0.77). When comparing resting-state seed and ICA images, the strongest DICE coefficients were obtained with the images $\tilde{A_{2}}$ for $HbO_{2}$ (DICE=0.60) and $\tilde{A_{1}}$ for Hb (DICE=0.77). For the patient 3, no ICA binary masks corresponded to those identified on the task-based maps. When comparing resting-state seed and ICA images, the strongest DICE coefficients were obtained with the images $\tilde{A_{3}}$ for $HbO_{2}$ (DICE=0.60) and $\tilde{A_{2}}$ for Hb (DICE=0.57).

## 4. Discussion

The results of this study showed that seed correlation and ICA resting-state methods are capable of identifying functional brain areas using intraoperative RGB imaging. Three patients were included in this study, including two patients in awake surgery and one patient under general anesthesia. It is interesting to note that the resting-state methods allow the identification of functional areas for the patient under general anesthesia and for the two patients who underwent awake surgery. However, the robustness and performance of these methods need to be evaluated on a larger number of patients. Intraoperative resting-state imaging has several advantages compared to task-based analyses: data acquisition is shorter, less complex, and less demanding for the patients, especially for those unable to perform the tasks.

The functional areas identified by the resting-state analyses present a strong similarity with those obtained with the task-based analysis. When comparing $HbO_{2}$ and Hb task-based maps to resting-state seed maps and resting-state ICA maps $\tilde{A_{4}}$$HbO_{2}$ and $\tilde{A_{2}}$Hb for patient 1, $\tilde{A_{2}}$$HbO_{2}$ and $\tilde{A_{1}}$Hb for patient 2 and $\tilde{A_{1}}$$HbO_{2}$ and $\tilde{A_{3}}$Hb for patient 3, the mean and standard deviation of the DICE coefficient are 0.55 and 0.29, respectively (this notation is indicated mean ± standard deviation in the rest of the manuscript). The large dispersion of the DICE coefficient is due to the low values computed for patient 3. For patients 1 and 2, the values of the DICE coefficient computed between the task-based maps and resting-state seed maps ($HbO_{2}$ and Hb maps) are included between 0.77 and 0.85 (DICE:0.81±0.03), which indicates a strong similarity between resting-state seed and task-based maps. These resting-state seed maps also present a strong overlap with the task-based maps (Overlap:0.76±0.11). For patient 3, the values of the DICE coefficient are very low (for $HbO_{2}$ and Hb maps, $DICE(tb,rs_{seed})\leq 0.12$) which indicates that the resting-state seeds maps do not match the task-based maps. Although the resting-state seed analysis makes it possible to identify a cortical area close to the motor area, the low values of the DICE coefficient are mainly due to the incorrect functional identification provided by the task-based analysis, which was not due to improper image acquisition. In order to increase the robustness of the task-based analysis, the patient’s Hb and $HbO_{2}$ response function to an impulse stimulus could be experimentally measured. These response functions are patient dependent and differ depending on the type of cortical tissue [39]. Moreover, the neurovascular system evolves with age, which implies a change in hemodynamic response [32] and the progression of gliomas over time implies a change in the hemodynamic response [23].

For patients 1 and 2, the resting-state ICA maps ($\tilde{A_{4}}$$HbO_{2}$ and $\tilde{A_{2}}$Hb for patient 1 and $\tilde{A_{2}}$$HbO_{2}$ and $\tilde{A_{1}}$Hb for patient 2) present a strong similarity with the task-based maps (DICE:0.69±0.06). This mean value is 15% lower than the one obtained with the resting-state seed maps, and the standard deviation is 133% higher than the one obtained with the resting-state seed maps. These differences are due to the detection of several other cortical networks using the ICA method, see Figure 3. These resting-state ICA maps also present a strong overlap with the task-based maps (Overlap:0.65±0.09). This mean value is 14% lower than the one obtained with the resting-state seed maps, and the standard deviation is 18% lower than the one obtained with the resting-state seed maps. For patient 3, the values of the DICE coefficient are very low (for $HbO_{2}$ and Hb maps, $DICE(tb,rs_{ICA})\leq 0.21$) which indicates that the resting-state seeds maps do not match the task-based maps. However, the resting-state ICA maps $\tilde{A_{2}}$ makes it possible to identify a cortical area close to the motor area. When comparing the resting-state seed and ICA analyses, we notice that the ICA maps $\tilde{A_{4}}$$HbO_{2}$ and $\tilde{A_{2}}$Hb for patient 1, $\tilde{A_{2}}$$HbO_{2}$ and $\tilde{A_{1}}$ *H*b for patient 2 and $\tilde{A_{3}}$$HbO_{2}$ and $\tilde{A_{2}}$Hb for patient 3 present a strong similarity with the resting-state seed maps (DICE:0.66±0.11). These results suggest that the resting-state seed and ICA analyses are more robust that the task-based analysis for identifying functional brain areas.

For the patient 2, the tumor is directly observable on the RGB image. The binary masks on images $\tilde{A_{2}}$Hb, $\tilde{A_{3}}$$HbO_{2}$ and $\tilde{A_{5}}$Hb are connected to the tumor. In these images, the resting-state ICA analysis seems to highlight patterns of spatial hemodynamic fluctuations close to the tumor and is likely to indicate an abnormal functional connectivity provoked by the tumor. Indeed, a fMRI resting-state study showed that abnormal functional connectivity could be detected not only adjacent to the visible tumor but also in distant brain tissue, even in the contralesional hemisphere [40]. Montgomery et al. [23] also demonstrated that tumor growth in an awake mouse brain disrupts the synchrony of both neuronal activity and hemodynamics, but did not directly assess the coupling relationship between neuronal activity and hemodynamics.

## 5. Conclusions

The resting-state seed method could be roughly represented as a “digital EBS” in the sense that the neurosurgeon has to select a portion of the cortex (seed) to identify the connected portion of tissue. However, the neurosurgeon must have an idea of the position of the functional area. In case the tumor has strongly displaced the functional areas, this method can be put in default. This issue could be addressed by using preoperative fMRI maps to automatically place the resting-state seeds. Thresholding operations (see Equation (4)) can be automated by using a linear general model combined with random field theory [41]. The optical resting-state maps could be projected on a three dimension neuroanatomy atlas, which would help to obtain a better understanding of the extent of the seed correlation and ICA maps. This would also allow to establish a comparison between optical resting-state maps and fMRI pre-operative maps. As opposed to fMRI and fNIRS resting-state analyses [17,33], the spatial patterns expressed in the ICA method cannot be sorted by comparing the patterns in the images to neuroanatomy atlas. This issue will be addressed in a future study by comparing optical and fMRI resting-state maps. The ICA method was implemented with 5 independent components. Li et al. [42] proposed a method to estimate the number of independent components for fMRI data, this method could be adapted to our data in future studies. In this feasibility study, resting-state maps were compared to task-based maps using the DICE and Overlap coefficients. The objective was to show that fMRI resting-state methods can be used to retrieve intraoperative resting state maps using RGB imaging. In a future study, a threshold value of the DICE and overlap coefficients could be defined to validate intraoperative resting-state maps against task based maps. For this purpose, a validated database containing task-based and resting-state functional maps is needed, which implies a larger number of patients, as well as a suitable methodology to compare the measured data with the database.

In this study, we present the methodology for the identification of resting-state networks using RGB imaging during neurosurgery. The detection of functional brain areas using the resting-state seed and ICA analyses presented a strong similarity with those identified with a task-based analysis. These areas corresponded to the identification provided by EBS when patients are awake and under general anesthesia. This work demonstrates that RGB imaging combined with quantitative modeling of brain hemodynamic biomarkers can robustly assess functional brain areas during patient rest and before brain tumor resection surgery. This reinforces the relevance of using conventional RGB imaging for intraoperative functional brain imaging.

## Figures and Tables

**Table 1:** Information on patients and acquisitions.

		Patient 1	Patient 2	Patient 3
Gender		Female	Male	Female
Age		37	57	45
Tumor		Low grade glioma	Lung cancer metastasis	Low grade glioma
Surgical window		Right hemisphere	Right hemisphere	Right hemisphere
General status		Awake	General anesthesia	Awake
Task-based analysis	Task	Left-hand movement	Left-hand movement	Left-hand movement
		performed by the patient	performed by an external person	performed by the patient
	Number of cycles	2	3	3
	Acquisition duration	1 min	2 min	2 min
Resting-state analysis	Patient status	Looked at a medical practitioner	Under general anesthesia	Looked at a medical practitioner
		and did not make any movements	and did not make any movements	and did not make any movements
	Acquisition duration	1 min 40 s	2 min 20 s	2 min 20 s

**Table 2:** DICE and overlap coefficients computed between task-based (tb) and resting-state seed ($rs_{seed}$) binary maps.

		Patient 1	Patient 2	Patient 3
$HbO_{2}$	$DICE(tb,rs_{seed})$	0.85	0.77	0.12
	$Overlap(tb,rs_{seed})$	0.86	0.64	0.11
Hb	$DICE(tb,rs_{seed})$	0.84	0.78	0.10
	$Overlap(tb,rs_{seed})$	0.89	0.65	0.09

**Table 3:** DICE and overlap coefficients computed between task-based (tb) and resting-state ICA ($rs_{ICA}$) maps. The DICE coefficients computed between resting-state seed ($rs_{seed}$) and ICA ($rs_{ICA}$) maps are also indicated.

		Patient 1					Patient 2					Patient 3				
		$\tilde{A_{1}}$	$\tilde{A_{2}}$	$\tilde{A_{3}}$	$\tilde{A_{4}}$	$\tilde{A_{5}}$	$\tilde{A_{1}}$	$\tilde{A_{2}}$	$\tilde{A_{3}}$	$\tilde{A_{4}}$	$\tilde{A_{5}}$	$\tilde{A_{1}}$	$\tilde{A_{2}}$	$\tilde{A_{3}}$	$\tilde{A_{4}}$	$\tilde{A_{5}}$
$HbO_{2}$	$DICE(tb,rs_{ICA})$	0.49	0.25	0.50	0.57	0.20	0.47	0.73	0.14	0.00	0.07	0.20	0.12	0.13	0.06	0.06
	$Overlap(tb,rs_{ICA})$	0.52	0.24	0.46	0.52	0.19	0.44	0.60	0.11	0.00	0.06	0.19	0.12	0.12	0.07	0.06
	$DICE(rs_{seed},rs_{ICA})$	0.46	0.31	0.49	0.52	0.20	0.55	0.87	0.15	0.00	0.03	0.46	0.35	0.60	0.10	0.23
Hb	$DICE(tb,rs_{ICA})$	0.59	0.74	0.06	0.14	0.26	0.75	0.00	0.05	0.51	0.07	0.15	0.16	0.21	0.08	0.20
	$Overlap(tb,rs_{ICA})$	0.70	0.70	0.06	0.14	0.25	0.77	0.00	0.04	0.41	0.06	0.15	0.15	0.18	0.08	0.18
	$DICE(rs_{seed},rs_{ICA})$	0.60	0.70	0.16	0.23	0.17	0.69	0.00	0.00	0.65	0.07	0.14	0.57	0.03	0.41	0.12

## Abbreviations

The following abbreviations are used in this manuscript:

fMRI	functional Magnetic Resonance Imaging
fNIRS	functional Near Infra-Red Spectroscopy
EBS	Electrical Brain Stimulation
$HbO_{2}$	oxy hemoglobin
Hb	deoxy hemoglobin
tb	task-based
rs	resting-state
ICA	Independent Component Analysis
